# Draft Genome Sequence of Heavy Metal-Resistant *Cupriavidus alkaliphilus* ASC-732^T^, Isolated from Agave Rhizosphere in the Northeast of Mexico

**DOI:** 10.1128/genomeA.01013-16

**Published:** 2016-09-22

**Authors:** Fernando Uriel Rojas-Rojas, Marcel Huntemann, Alicia Clum, Manoj Pillay, Krishnaveni Palaniappan, Neha Varghese, Natalia Mikhailova, Dimitrios Stamatis, T. B. K. Reddy, Victor Markowitz, Natalia Ivanova, Nikos Kyrpides, Tanja Woyke, Nicole Shapiro, J. Antonio Ibarra, Paulina Estrada-de los Santos

**Affiliations:** a Instituto Politécnico Nacional, Escuela Nacional de Ciencias Biológicas, Prol. Carpio y Plan de Ayala s/n, Col. Santo Tomás, Del. Miguel Hidalgo, Ciudad de México, Distrito Federal, México; b DOE Joint Genome Institute, Walnut Creek, California, USA

## Abstract

*Cupriavidus alkaliphilus* ASC-732^T^ was isolated from the rhizosphere of agave plant growing in alkaline soils in San Carlos, Tamaulipas, Mexico. The species is able to grow in the presence of arsenic, zinc, and copper. The genome sequence of strain ASC-732^T^ is 6,125,055 bp with 5,586 genes and an average G+C content of 67.81%.

## GENOME ANNOUNCEMENT

Strain ASC-732^T^ was isolated from agave rhizosphere (1) and described later as *Cupriavidus alkaliphilus* (2). The draft genome of *Cupriavidus alkaliphilus* ASC-732^T^ was generated at the DOE Joint Genome Institute (JGI) using Illumina technology (3). An Illumina standard shotgun library with 300-bp inserts was constructed and sequenced using the Illumina HiSeq 2500 1-TB platform, which generated 7,956,522 reads totaling 1,193.5 Mbp. All general aspects of library construction and sequencing performed at the JGI can be found at http://www.jgi.doe.gov. All raw Illumina sequence data were filtered using BBDuk (Bushnell), which removes known Illumina artifacts, and PhiX. Reads with more than one “N” or with quality scores (before trimming) averaging less than 8 and reads shorter than 51 bp (after trimming) were discarded. The remaining reads were mapped to masked versions of human, cat, and dog references using BBMAP (4) and discarded if identity exceeded 95%. Sequence masking was performed with BBMask (4). The following steps were then performed for assembly: (a) artifact-filtered Illumina reads were assembled using SPAdes version 3.6.2 (5); (b) assembly contigs were discarded if length was <1 kbp. The final draft assembly contained 36 contigs in 34 scaffolds, totaling 6.125 Mbp in size and was based on 1,193.5 Mbp of Illumina data with a mapped coverage of 194.5×.

After individual searches using the IMG/MER BLAST tool, a number of genes involved in functions such as motility (flagella), chemotaxis, adhesion (type I-like fimbrae), biofilm formation (PGA synthesis), and multiple secretion systems were found: one Sec-like; one set each of type I, III, and VI; and three sets of type II, which are likely encoding for type IV pilus systems. Also present are genes for resistance to nickel, zinc, arsenate, chromate, cobalt, cadmium and copper; the P-subunits for phenol degradation; and genes for the production of secondary metabolites such as bacteriocins, nonribosomal synthesized peptides, siderophores, and polyketides. The average G+C content is 67.81%, which is very similar to the one reported during the original description (66.8%).

### Accession number(s).

The draft genome sequence was deposited in the European Nucleotide Archive at EMBL-EBI under the accession numbers FMAD01000001 to FMAD01000034. The BioProject accession number at NCBI is 329738.
